# Contribution of Laboratory Centralisation to Delays in Blood Culture Handling and Determination of Results

**DOI:** 10.7759/cureus.115480

**Published:** 2026-08-31

**Authors:** Malila R Noone

**Affiliations:** 1 Microbiology, Independent Researcher, Darlington, GBR

**Keywords:** antimicrobial resistance, blood cultures, broad-spectrum antibiotic therapy, laboratory centralisation, laboratory costs, laboratory quality, pathology laboratory report interpretation, preanalytical phase of the laboratory, rapid microbial diagnostics, surviving sepsis guidelines

## Abstract

Introduction and aims: Early clinical diagnosis and prompt initiation of antimicrobial therapy have been widely recognised as being crucial to survival from sepsis, but the critical supportive role played by clinical microbiology services is less well recognised. Minimising pre-analytical delay of blood cultures yields early results which support the effective and targeted management of sepsis, while delayed results lead to the prolonged use of empiric broad-spectrum antimicrobial therapy which is considered a causal factor in the emergence of antimicrobial resistance. This study is limited to England and aims to study compliance with a four-hour, pre-analytical delay standard by clinical microbiology services. The study further aims to appraise the impact of laboratory centralisation on microbiology services.

Methods: This is a retrospective observational study over a single year. Retrospective audit data was obtained through freedom of information requests. Information regarding service configurations and costs during that period was also obtained and supplemented by data available in the public domain.

Results: Data relating to 149 acute hospitals showed poor rates of compliance, with only 2.7% (n=4) showing full compliance with the recommended standard. Compliance rates showed wide variations between hospitals across the region and within the same administrative unit. Service configurations were not standardised, and quality requirements were not met. Anticipated savings were not realised.

Conclusions: Poor rates of compliance with the pre-analytical delay standard are likely to adversely affect patient outcomes from sepsis and impact on antimicrobial stewardship. Laboratory centralisation strategies failed to address the particular needs of the microbiology service. Remedial action is required, with a view to establishing a more comprehensive microbiology service in each acute care setting. Changes are justified in view of the continued high mortality associated with sepsis and the increasing emergence of antimicrobial resistance.

## Introduction

Sepsis is a complication of infection which is associated with high mortality rates and life-changing after-effects. It has gained widespread public attention through initiatives such as the Surviving Sepsis Campaign and other awareness campaigns [1,2], with increasing rates of sepsis also being reported [3]. The clinical diagnosis of sepsis is based on the International Consensus guidelines [4], and prompt initiation of antimicrobial therapy following clinical diagnosis is crucial for patient survival. International guidelines also recommend blood culture as the gold-standard diagnostic test, as it can confirm the presence of infection and identify the causative pathogen [5]. Although the current practice of administering broad-spectrum antimicrobial agents on an empirical basis has proved to be effective [6,7], identification of the infective agent and determination of antimicrobial susceptibilities is invaluable. It may instigate a lifesaving switch to more effective agents if initial empirical therapy is deemed to be sub-optimal. The likelihood of this is increasing, with the growing prevalence of antimicrobial resistance (AMR) [8]. The emergence of AMR has been linked to the use of broad-spectrum antimicrobial therapy, which, although vital, is often unduly prolonged. Timely susceptibility reports support early de-escalation of broad-spectrum therapy to the most effective narrow-spectrum agents, which is strongly advocated in current antimicrobial stewardship programs [9].

Current continuous-monitoring automated blood culture systems show increased sensitivity and can provide provisional reports within 8-18 hours of procuring a blood culture if collection and management of the specimen is optimised [10]. A delay between specimen collection and incubation in the analyser (pre-analytical delay) can prolong turnaround times, hinder timely antimicrobial de-escalation, and consequently contribute to the emergence of antimicrobial resistance (AMR) and other iatrogenic harms [11]. A negative result promotes withdrawal of antimicrobials altogether but runs the risk of being discounted unless it is validated by a record of optimal specimen quality and minimal pre-analytical delay. Early identification of the pathogen has added clinical benefits. It may point to the probable source of infection and, since mortality risk and clinical progress vary across organisms, it also serves as a prognostic indicator guiding the use of adjunctive therapeutic agents and clinical interventions [12]. When reports of pathogens and their susceptibilities are expedited, there is a wider impact, such as on mortality rates and health care costs [13]. In summary, timely blood culture results support the effective management of sepsis and limit the emergence of AMR. 

Minimising pre-analytical delay (PAD) not only provides early results: it also increases detection rates. Although pathogen survival in blood culture media is variable and affected by many factors such as species characteristics and holding temperatures, the effect of delay on survival is recognised as an important controllable factor leading to the proposal of pre-analytical delay standards [14]. Pre-analytical delay, simulated as occurring outside laboratory operating hours, has been shown to result in a reduced yield [15,16]. It has even been suggested that the delay in handling blood cultures taken over the weekend when laboratory services were reduced may be linked to the increased mortality observed over the weekend and branded the weekend effect [17]. These reports highlight the impact of microbiology service schedules on blood culture results and patient outcomes. Modification of service configurations and schedules which occurred during laboratory centralisation in England may have had an unanticipated impact on the quality of National Health Service (NHS) microbiology services. 

The NHS is a publicly funded healthcare system in the United Kingdom (UK) comprising devolved services in Scotland, Wales and England. In 1990, acute care hospitals in England became independent administrative units termed trusts. Each Trust was responsible for providing laboratory services for patients in hospital as well as a community service for primary care General Practitioners (GPs) in a designated geographical area. When Trusts subsequently merged, a laboratory was retained in each hospital.

In 2006, an Independent Review found England's NHS pathology service to be one of the best value systems in the world with expenditure per capita on in-vitro diagnostics about half that of equivalent countries in Europe, and a quarter of that in the USA [18]. Nevertheless, in 2008, back office and pathology services (categorised as being comparable) were earmarked for speedy centralisation [19]. Centralisation was driven by the potential for savings, with a target reduction of global pathology expenditure (expenses for all pathology sub-specialities) from approximately 4% of total trust expenditure to <1.6%. The hub and spoke model recommended was expected to achieve benefits through economies of scale by concentrating non-urgent work at a centralised hub. In spoke hospitals, essential services laboratories (ESLs) or hot labs provided only urgent blood tests, which were defined as time-critical and requiring an analytical turnaround time (TAT) of four hours. Microbiology specimens were deemed non-urgent and spoke hospitals transferred them to a hub while retaining their clinical (usually medically qualified) microbiologists on site. Although analytical TATs of microbiological specimens range from three to five days, they are also time-critical and international guidelines recommend they are processed as soon as possible [20]. The diversity of infectious agents and specimen types precludes precise time limits for handling microbiological specimens and leaves the recommendation as soon as possible open to interpretation or disregarded altogether. This was a cause for concern and was considered a step too far when centralisation of microbiology services was introduced in England [21].

The United Kingdom Standards for Microbiology Investigations (UKSMI) now recommends incubating inoculated blood cultures in an automated analyser as soon as possible and within four hours of collection [14]. This study documents compliance with this standard by hospitals in England for the financial year 2022-23. The changes accompanying centralisation and their impact on microbiology services are appraised. Wider clinical implications and governance issues are also examined, and future direction considered.

## Materials and methods

This is a retrospective observational study documenting compliance with the four-hour, pre-analytical standard for blood cultures by NHS Trusts in England.

Data collection

Freedom of Information (FOI) requests were submitted to all acute-care non-specialist Trusts which were listed in the NHS England provider directory (Appendix A). The submission was addressed to the designated FOI officer in each of 116 Trusts, with the expectation of collaboration with the Trust's clinical microbiologist. The principal enquiry related to the percentage of blood culture sets incubated within four hours of specimen procurement at each acute hospital within the Trust in the financial year 2022-23. Additional questions helped outline microbiology service configurations, and responses were supplemented by data available in the public domain. The standard opening hours of each laboratory were not requested, as compliance with the PAD standard only relates to incubation facilities rather than specimen processing facilities. Global pathology cost as a percentage of total Trust costs for the same year was also requested. The Trust annual financial reports are available to the public, but the cost of the laboratory service is usually included in support service costs and not specified.

The UK Freedom of Information Act 2000 provides a right of access to information held by public authorities including the NHS, and Trusts are legally obliged to respond to requests. A public body may refuse to release information which is deemed commercially sensitive, is not readily available or can be found elsewhere, but any information released in response to a FOI request is deemed to be factually correct. The information, once released, is in the public domain and may be shared with appropriate safeguards. The data reported here do not contravene copyright restrictions and personal data protection guidance and preclude identification of individual trusts. Further data are withheld in order to prevent identification of individual trusts. Ethical approval was not required as patient-identifiable data was not requested.

Statistical methods

Numerical data are presented as counts and percentages. Differences between compliance rates achieved vs the expected rates were analysed with a χ2-test. To assess differences in the costs of blood culture services across different configuration categories, the data were first tested for normality, followed by the application of the Kruskal-Wallis test. A p-value <0.05 was considered statistically significant.

## Results

Of the 116 trusts approached, there was no reply from 12.9% (n=15). They were not pursued due to awareness that administrative changes and clinical service emergencies may prevent prompt responses to FOI requests. A further 10.3% (n=12) trusts stated that they did not have the data requested and suggested referral of the questionnaire to their off-site service provider. Since an off-site provider would not be responsible for the pre-analytical management of specimens, an appeal was made for the trust clinical microbiologist to be made aware of the FOI request. This did not change the stand taken by these trusts and there was no response to the questionnaire. Of the 116 trusts sampled, data were supplied by 76.7% of trusts (n=89). All data received are presented and analysed.

Service configurations

Data are presented for 89 trusts managing 146 acute hospitals. Acute hospitals were those providing 24-hour acute-care/emergency services and submitting more than 500 blood cultures annually. Service configurations varied widely (Table 1).

**Table 1:** Microbiology service configurations in trusts (n=89) and hospital (n=146).

Configuration Category	Trusts (n)	Acute hospitals n	Hubs or hospitals with on-site incubation and culture n (%)	Spoke hospitals n (%)
A: Single trusts, 1-2 hospitals per trust, on-site incubation and culture in each hospital	31	36	36 (24.6%)	0
B: Single trusts, 2-6 hospitals per trust, hub and spoke within trust	29	69	32 (21.9%)	37 (25.3%)
C: Inter-trust networks, 1-4 hospitals per trust, hub & Spoke configuration	25	35	15 (10.2%)	20 (13.6%)
D: Single trusts, 1-2 hospitals per trust, off-site commercial hub	4	6	(3 non-hospital sites)	6 (4.1%)
Total	89	146	83 (56.8%)	63 (43.1%)

Standard arrangements for incubation and processing of blood cultures on-site in each hospital had been retained by 38% of trusts (n=31, Group A). Hub and spoke configurations applied to 65% of trusts (n=58, Groups B, C, D). These arrangements were either within their own trust, as inter-trust networks, or by using commercial off-site providers.

Arrangements for incubation at spoke hospitals

Of 146 hospitals, 43.1% were spoke hospitals (n=63). Arrangements for incubation of blood cultures by the spoke hospitals are shown in Table 2. 

**Table 2:** Blood culture incubation arrangements in spoke hospitals (n=63).

Configuration category	Spoke with satellite unit, on-site and positives to hub n (%)	Spoke with transport, direct to hub n (%)	Total spokes n
A: Single trusts, 1-2 hospitals/trust on-site incubation and culture in each hospital	NA	NA	NA
B: Single trusts, 2-6 hospitals/trust, hub and spoke within trust	11 (17.4%)	26 (41.2%)	37
C: Inter-trust networks, 1-4 hospitals/trust, hub and spoke	9 (14.2%)	11 (17.4%)	20
D: Single trusts, 1-2 hospitals/trust, off-site commercial hub	4 (6.3%)	2 (3.1%)	6
Total	24 (38%)	39 (62%)	63

Satellite units for on-site incubation were sited in 38% of spoke hospitals (n=24), and only bottles flagging positive were transported to the hub laboratory to be processed. In one of these spoke hospitals, specimens flagging positive within 18 hours were tested using a BioFire Blood Culture Identification Panel before transfer to the hub. Spoke-to-hub distances for the 63 spoke hospitals ranged from 1.9-40 miles (median 10miles).

Compliance with the pre-analytical delay standard

Compliance data was measured on at least one occasion in 59.5% of trusts (n= 53) and only 15.7%) (n= 14) reported undertaking regular audit. There were disparities in compliance rates between hospitals in a single trust and between hospitals within a single inter-trust network. Compliance is reported as the percentage of blood cultures incubated within four hours with compliance of 100% indicating that all blood cultures were incubated within 4 hours. Compliance rates are presented for the 146 hospitals (Table 3).

**Table 3:** Percentage compliance with pre-analytical standards by hospitals n=146.

	100% n (%)	80-99% n (%)	50-79 n (%)	<50 n (%)	No data n (%)	Total n
Hubs or on site culture (n)	3	24	25	9	22	83
Spokes with satellite unit (n)	1	12	3	4	4	24
Spokes direct transport (n)		6	4	19	10	39
Total (n)	4 (2.7%)	42 (28.7%)	32 (21.9)	32 (21.9%)	36 (24.6%)	146

Full compliance was reported by only 2.7% of hospitals (n=4). A further 28.7% (n=42) reported a>80% compliance rate. This rate of compliance is suggestive of incubation of cultures beyond routine laboratory hours or the use of satellite units, as it was reported that when incubation was restricted to routine laboratory hours, only approximately 50% of specimens achieved incubation within the four-hour standard. A compliance rate of 80% was shown by 31.5% of hospitals (n=46), which is also significantly below satisfactory compliance(p>1).

Compliance data for 2022-23 was not available for 40.4% of trusts (n=36). Reasons given for the lack of data included inability to access or interrogate the computer system and inability to change computer settings. Problems included the inability to record the time of specimen procurement, although this is a legal requirement and recorded on both the request form and specimen label. The interval between time of specimen procurement and time of incubation was not routinely included in culture reports. Several trusts reacted when delays were noticed, but only one trust reported inclusion of an explicit comment in the final report indicating that the blood culture was received in the laboratory more than four hours after it was taken and that the delay could reduce the chance of growing relevant pathogens.

Cost of the laboratory service as a percentage of trust cost

Costs were provided in 79.7% of trusts (n=71). Cost data were withheld by 20.2% (n=18), citing it as commercially sensitive or confidential (Table 4).

**Table 4:** Costs by configuration: interquartile range (IQR) and median.

Configuration Category	Trusts n	Costs withheld n (%)	Costs reported n (%)	IQR/median
A: On-site incubation and culture	31	3	28	2.9-4.0/3.19
B: Hub and spoke within trust	29	6	23	3.0-4.6/3.3
C: Hub and spoke inter-trust	25	8	17	3.0-4.7/3.7
D: Off-site commercial	4	1	3	N/A
Total	89	18 (20.2)	71 (79.7%)	2.95-4.31/3.3

There are no significant differences between the mean ranks of any pair (p=0.67-0.9). In particular, hub and spoke arrangements (median 3.3 and 3.7) did not appear to confer a cost advantage over trusts which had retained on-site incubation and culture (median 3.19).

## Discussion

The early clinical diagnosis of sepsis and the prompt initiation of antimicrobial therapy have been widely recognised as being crucial to survival. The critical role played by clinical microbiology services in supporting the management of sepsis and reducing the emergence of antimicrobial resistance is less well recognised and has not been monitored. This report is unique in that it relates to microbiology services across a single region served by a single healthcare provider.

Compliance with the recommended four-hour standard, which minimises the pre-analytical delay of blood cultures, is an auditable quality measure of a microbiology service and reflects its ability to support the effective management of sepsis. This study shows that only 2.7% (n=4) of 146 hospitals in England showed full compliance with this key quality indicator. A further 28.7% (n=42) showed >80% compliance. It may be argued that this rate of compliance is acceptable, but it is significantly low and less than ideal. 

The impact of laboratory centralisation on the ability to comply with quality standards, and the impact on wider healthcare and governance issues can be assessed using the incidental data obtained in response to the FOI questionnaire. The issues highlighted below relate in particular to pre-centralisation planning and the attention given to the particular requirements of the microbiology service.

Service configurations

Although some variation in service configuration is expected due to geographical factors and the distribution of urban centres, the marked differences observed in this study suggest a lack of a cohesive strategy following the implementation of centralisation. The disparities in quality standards between hospitals in the region, and often within a single management entity, further indicate that decisions relating to the service and quality targets were left to individual trusts. Centralisation of laboratory services has been undertaken in many countries, and a core strategy with an obligatory quality framework facilitating prospective review of post-centralisation services has been undertaken elsewhere [22]. 

Hub and spoke systems

This recommended system applied to 65% (n=58) of the 89 trusts providing data. Sixty-three spoke hospitals had suffered the loss of their on-site microbiology laboratories and their specialist biomedical scientists (BMS). Hypothetically, some of the financial savings thus released might have been available for investment in robust transport facilities and enhanced out-of-hours (OOH) services. There does not, however, appear to have been a mandate for this. Specimen transport arrangements appear less than adequate as, of 39 spoke hospitals relying on transport, only 15.3% (n=6) showed >80% compliance. Limited enhancement of OOH services and >80% compliance was seen in 28.7% of hospitals (n=42). As only 9% of attendances at NHS emergency centres occur between the hours of midnight and 7 am, with a fall in blood culture numbers during these hours, personal experience has shown that 80% compliance can be achieved by a single negotiated attendance by a BMS between 10 pm and midnight. 

Information technology and audit

Regular audit of procedural and quality standards is recognised as good laboratory practice by the Royal College of Pathologists (RCPath) [23]. Although laboratory centralisation was accompanied by investment in information technology (IT) infrastructure, auditable standards for microbiology do not appear to have been incorporated into laboratory systems. There was no compliance data for 2022/23 for 40.4% of trusts (n=36), and only 15.9% (n=14) reported an ability to undertake regular audits. The inherent complexity and changing needs of microbiology present a challenge, and resolution is more problematic when IT support is off-site or outsourced.

Savings

Centralisation was driven by the demand for cost savings, but the target reduction of pathology expenditure to <1.6% of trust expenditure has not been realised. Although further examination will be required to analyse the distribution of costs by department, the potential contribution of the microbiology laboratory to the savings agenda is likely to be limited. Centralising serological specimens which benefit from automation promises savings, but costs arising from transport, bio-hazard regulations and tracking suggest this should be limited to the more complex investigations. Economies of scale do not readily translate to specimens needing labour-intensive manual procedures, and transport delays affect the quality of these specimens. The NHS is able to take advantage of discounts associated with bulk purchasing and leasing agreements across trusts and, except in the case of specialised investigations, should be able to retain microbiology tests on site. It is noteworthy that hub and spoke arrangements did not appear to confer a cost advantage over trusts which had retained microbiology services on-site. 

Governance and quality management

Although laboratory centralisation has been undertaken in many countries in pursuit of financial savings, there are few reports of its impact on subsequent microbiology service performance and quality [22]. Responsibility for the quality of the service clearly lies with the designated hospital clinical microbiologist, and this has ethical implications [24]. Referral of the FOI request to the off- site service provider on 12 occasions in this report hints at a lack of direct control, a lack of ownership, disengagement with quality issues or a sense of alienation following the move of the hospital laboratory service off-site. The lack of mandatory status for UK SMIs may also have disempowered clinical microbiologists and curtailed their ability to secure the investment and support required to upgrade their service. Minimising PAD does not lie solely with the laboratory. Pre-analytical management of specimens is a clinical issue, and the clinician requesting a blood culture must recognise the need to transport the specimen immediately for prompt incubation. The clinical microbiologist is identified as being responsible for facilitating this, for education of clinicians and for monitoring compliance by performance of quality audits.

Wider aspects of healthcare

There have been no proactive measures in place to monitor the impact of laboratory centralisation on wider aspects of healthcare such as duration of stay, antibiotic use, healthcare-associated infections and the emergence of antimicrobial resistance. Although healthcare outcomes are reported by trusts, they are rarely delineated by hospital, and there is no reference to the local configuration of microbiology services. Consequently, iatrogenic patient harms, sepsis survival and the emergence of AMR may not be recognised as attributable to laboratory shortcomings due to centralisation. Elsewhere, the impact of microbiology laboratory centralisation on antimicrobial stewardship and hospital-acquired infections and their associated costs has been reviewed [25-27], and the need for a more comprehensive service in each acute care setting has been rationalised.* *

Limitations of recent modifications

A limited reversal of centralisation has been undertaken by the installation of satellite units in several hospitals which had lost on-site microbiology facilities. Prompt incubation of blood cultures is, however, only the first step in the pathway to providing speedy results. If positive bottles are not processed swiftly, delays in time to Gram stain and time to antimicrobial test results will continue to cause patient harm [28]. An adequate response to the Surviving Sepsis campaigns requires a seamless and speedy pathway from specimen collection to the final report. The use of molecular tests on positive bottles and on EDTA blood samples will speed up the management of sepsis but only in relation to the most common pathogens. These new diagnostic systems do not support centralisation of microbiology facilities as they require effective on-site clinical liaison, which is more difficult when the service is centralised [29]. Furthermore, new technologies cannot entirely replace standard diagnostic microbiology, which is critical for the detection of rare and emerging pathogens and unusual antimicrobial resistance patterns. In addition to blood cultures, specimens from associated sites also warrant prompt attention as they may yield the only positive isolate from a patient with sepsis. National good practice recommendations and associated standards for all procedures have been formulated.

Limitations of the study

This study is retrospective, but the data presented are results formally documented during the previous year and do not rely on memory. Although presented as reported data, the use of a freedom information enquiry ensures that the responses provided are factually correct. The results presented do not reflect changes in service configuration and improvement in compliance rates which are likely to have occurred following this study. The presentation is limited to microbiology services in England, but the principles addressed apply to most other countries. Although centralisation of laboratory services has been undertaken worldwide, there have been few reports of its impact on the quality of microbiology services.

This article was previously posted to the medRxiv preprint server on June 27, 2026 [30].

## Conclusions

Appraisal of the changes which accompanied laboratory centralisation in England shows that the particular needs of a microbiology service were not met. These changes hinder the optimal management of blood cultures, which in turn is likely to impact on the management of sepsis. Laboratory centralisation has been implemented in many countries with perceived benefits, but there have been similar concerns about its effect on microbiology services, and the need for a more comprehensive service in each acute care setting has been rationalised. The introduction of hot labs for urgent blood tests has been widely welcomed. Microbiology too should be recognised as an essential 24-hour, readily accessible service supporting wider clinical needs, control of infection requirements, antimicrobial stewardship, and the ability to respond to national demands such as outbreaks of infectious disease. Urgent changes should be considered in the context of the continued high prevalence of sepsis, and the emergence of antimicrobial resistance. Mandatory institution of good practice recommendations and standards is likely to be cost-effective if the impact on patient survival and indirect savings in health care costs are taken into account. There will also be considerable community benefit in terms of containing the emergence of antimicrobial resistance.

## Appendices

Appendix A 

Freedom of information (FOI) request questionnaire about blood cultures

1. Please list the hospitals or other acute sites currently submitting blood cultures and for each site;

i. Are blood cultures incubated and/or processed on site or sent to a microbiology laboratory off site?

ii. If incubated or processed off site, what is the distance in miles between the site and the off-site laboratory?

iii. How many blood culture sets were submitted from each site in 2022-23?

iv. Number of bed days for all patients per site.

**Table 5:** Table of the hospitals or other acute sites currently submitting blood cultures.

	Name of acute site	Blood incubated on- site? Y/N	Blood processed on site? Y/N	Distance to off-site lab in miles	No. cultures submitted 2022-2023 Adults and paeds	Number of bed days 2022-23
1.						
2.						
3.						
4.						

Comments

2. Does the trust follow the UK Standards for Microbiology Investigations (SMI S12) in relation to blood cultures?   YES/NO

3. Do you undertake formal compliance audits of the recommended pre-analytical standard for blood cultures of a four-hour TAT from collection to incubation?   YES/NO

4. What percentage of blood culture sets achieved this standard in the year 2022-23 at each site? If full compliance with the pre-analytical standard cannot be met, are clinicians made aware of the risk via a statement in the report on specimens which encountered delay? 

**Table 6:** Percentage of blood culture sets achieving this standard in the year 2022-23 at each site.

	Name of site	% achieving 4-hr TAT	Delayed incubation reported Y/N
1.			
2.			
3.			
4.			

Comments

5. What is the population served by the trust?                                                                                                          

6. What was the total expenditure on laboratory services (directorate of pathology) as a percentage of total trust expenditure in the year 2022-23? 

7. Please append a copy of your blood culture protocol or policy or other document which informs clinical staff when they should take a blood culture.
